# Increased CCL2, CCL3, CCL5, and IL-1β cytokine concentration in piriform cortex, hippocampus, and neocortex after pilocarpine-induced seizures

**DOI:** 10.1186/s12974-015-0347-z

**Published:** 2015-07-02

**Authors:** Gabriel M. Arisi, Maira L. Foresti, Khurshed Katki, Lee A. Shapiro

**Affiliations:** Texas A&M University Health Science Center, Department of Surgery, Temple, TX 76504 USA; Baylor Scott and White Health Care, Department of Neurosurgery, Temple, TX 76504 USA; Central Texas Veterans Health Care System, Temple, TX 76504 USA; Present address: Department of Physiology, Escola Paulista de Medicina, Universidade Federal de São Paulo, Rua Pedro de Toledo 669, Lab 3A, São Paulo, SP 04039-032 Brazil

**Keywords:** Chemokine, Cytokine, Interleukin, Epilepsy, Seizure, Inflammation, Multiplex

## Abstract

**Background:**

Cytokines and chemokines play an important role in the neuroinflammatory response to an initial precipitating injury such as status epilepticus (SE). These signaling molecules participate in recruitment of immune cells, including brain macrophages (microglia), as well as neuroplastic changes, deterioration of damaged tissue, and epileptogenesis. This study describes the temporal and brain region pattern expression of numerous cytokines, including chemokines, after pilocarpine-induced seizures and discusses them in the larger context of their potential involvement in the changes that precede the development of epilepsy.

**Findings:**

Adult rats received pilocarpine to induce SE and 90 min after seizure onset were treated with diazepam to mitigate seizures. Rats were subsequently deeply anesthetized and brain regions (hippocampus, piriform cortex, neocortex, and cerebellum) were freshly dissected at 2, 6, and 24 h or 5 days after seizures. Using methodology identical to our previous studies, simultaneous assay of multiple cytokines (CCL2, CCL3, CCL5, interleukin IL-1β, tumor necrosis factor (TNF-α)), and vascular endothelial growth factor (VEGF) was performed and compared to control rats. These proteins were selected based on existing evidence implicating them in the epileptogenic progression. A robust increase in CCL2 and CCL3 concentrations in the hippocampus, piriform cortex, and neocortex was observed at all time-points. The concentrations peaked with a ~200-fold increase 24 h after seizures and were two orders of magnitude greater than the significant increases observed for CCL5 and IL-1β in the same brain structures. TNF-α levels were altered in the piriform cortex and neocortex (24 h) and in the hippocampus (5 days) after SE.

**Conclusions:**

Pilocarpine-induced status epilepticus causes a rapid increase of multiple cytokines in limbic and neocortical regions. Understanding the precise spatial and temporal pattern of cytokines and chemokine changes could provide more viable therapeutic targets to reduce, reverse, or prevent the development of epilepsy following a precipitating injury.

## Findings

### Introduction

Secretion of cytokines is a fundamental component of the inflammatory response in the nervous system [1–4]. These proteins are important immunomodulators, and chemokines, a special class of cytokines, also possess chemoattractant properties. Following an initial precipitating injury such as status epilepticus (SE), alterations occur to the milieu of these neuroinflammatory proteins. Of the numerous functional outcomes attributed to these alterations, neuroplasticity has been implicated to play a prominent role in the epileptogenic progression. Other changes that have also been implicated in pathogenesis include vascular permeability, angiogenesis, and immune responses that contribute to tissue damage [2]. Considering that SE increases subsequent seizure susceptibility and the likelihood of developing epilepsy, immunomodulatory signaling proteins are garnering great interest as therapeutic targets to treat neuropathology and the epileptogenic progression [1, 3]. Further evidence in support of the potential for these proteins as therapeutic targets is found in patients with epilepsy. In these patients, studies have observed increased levels of chemokines CCL2, CCL3, and CCL5 (among others) in the hippocampus, other temporal lobe structures, and neocortex [4]. Moreover, elevated cytokines such as interleukin 6 (IL-6) and tumor necrosis factor (TNF) can be detected in serum [1], and previous experimental studies in rodents have yielded promising results regarding the potential therapeutic efficacy of targeting these particular inflammatory molecules. Considering these data, a greater understanding of the temporal and spatial changes of these proteins can yield more efficacious treatment options, as well as more precise targeting.

After the acute and early phases of SE, the brain typically enters into a latent period that precedes the development of spontaneous seizures. During this period, it has been suggested that neuroplastic and neuroinflammatory changes provide a substrate for hyperexcitable neural networks [5, 6]. Indeed, our previous studies have implicated the neuroinflammatory response in the hippocampus to provide a substrate for the aberrant sprouting of basal dendrites from immature and mature granule cells [7]. Considering the complex interactions that occur among the cytokines both in immune and nervous systems, the most efficacious method for examining their presence in epileptogenesis is through simultaneous multiplex immunoassay [8]. We previously demonstrated elevated hippocampal concentrations of chemokine C-C motif ligand 2 (CCL2) during the acute phase after SE [7]. This report extends these previous findings to further identify acute and early alterations to cytokine concentrations in the hippocampus, cerebellum, and piriform cortex, brain structures that are known to be involved in seizures and epilepsy.

## Methods

### Animals and seizure induction

Male Sprague-Dawley rats, weighing 230–235 g at the beginning of experiments were used in this study. The experimental procedure was approved by the IACUC of the Texas A&M University Health Science Center (protocol #2008-002-R).

SE was induced as previously described [7]. Animals were treated with pilocarpine hydrochloride in a single dose of 320 mg/kg i.p. or saline. The onset of SE was considered when animals exhibited a class 3 motor seizure in Racine’s scale of limbic seizures [9]. Only animals that experienced stage 5 seizures were used for analysis (*n* = 48). Ninety minutes after SE onset, rats were treated with diazepam (10 mg/kg) to mitigate seizures; control animals also received diazepam.

### Tissue harvest

Animals were deeply anesthetized using Euthasol (pentobarbital sodium and phenytoin, 1 ml/kg) and decapitated 2, 6, and 24 h or 5 days (*n* = 12 per time-point) after diazepam treatment. The brain was rapidly removed from the skull, and the hippocampus, cerebellum, piriform cortex, and a large slice of neocortex close to the midline containing the retrosplenial, cingulate, and primary motor cortex were dissected and immediately frozen.

### Cytokine analyses

Simultaneous measuring of different cytokines in brain regions was performed as previously described [8]. The following cytokines were assayed: CCL2, CCL3, CCL5, IL-1β, TNF-α, and vascular endothelial growth factor (VEGF) (Table 1) in tissue homogenates using a MILLIPLEX MAP kit (Millipore, Billerica, MA, USA) on a BIOPLEX 200 analyzer (BioRad, Hercules, CA, USA). The tissue concentration of each analyte was normalized to the total protein concentration measured with a Bradford assay and presented as a proportion of cytokines in picograms (pg) per microgram (μg) of total protein ± SEM. Student’s *t* test was employed to detect statistical differences in values of control and experimental groups.

**Table 1:** Average cytokine concentration in pg/μg of total protein

Analytes		Brain region	2 h		6 h		24 h		5 days	
Cytokines	Chemokines		Control	SE	Control	SE	Control	SE	Control	SE
	CCL2	Piriform	3.6	10.7	3.7	34.8	3.7	694.8	3.6	22.8
		Hippocampus	3.7	16.3	4.4	81.6	2.2	243.8	4.4	26.8
		Neocortex	2.9	21.7	3.5	97.0	3.7	933.3	3.8	15.5
		Cerebellum	1.4	1.8	1.4	1.9	2.8	4.0	3.8	5.7
	CCL3	Piriform	0.4	24.3	1.7	29.8	0.2	50.3	0.3	15.6
		Hippocampus	0.5	31.4	1.2	30.6	0.2	42.2	0.1	29.3
		Neocortex	0.9	32.3	0.9	23.3	0.1	37.7	0.3	22.1
		Cerebellum	0.2	0.2	0.0	0.0	0.0	0.9	0.0	0.0
	CCL5	Piriform	6.7	8.2	8.1	10.3	6.0	13.8	6.6	10.1
		Hippocampus	4.9	5.8	5.8	5.9	6.6	10.6	4.5	6.7
		Neocortex	8.6	8.9	7.9	10.5	8.2	14.2	8.4	8.2
		Cerebellum	7.1	5.9	7.1	6.2	7.2	6.0	6.8	5.8
IL-1β		Piriform	5.0	7.7	5.2	7.8	6.2	12.2	6.1	6.7
		Hippocampus	5.5	8.5	6.0	8.5	5.9	7.7	5.8	6.4
		Neocortex	6.4	10.0	5.6	9.1	5.7	13.4	6.4	8.0
		Cerebellum	6.0	6.4	5.0	5.7	5.3	6.5	5.7	6.7
TNF		Piriform	1.3	1.0	0.7	0.8	1.2	0.6	1.1	1.6
		Hippocampus	1.6	1.6	1.3	1.9	2.5	1.6	1.5	2.8
		Neocortex	1.2	1.5	1.0	1.5	0.7	1.5	2.0	1.5
		Cerebellum	1.0	1.0	0.5	0.4	0.6	0.5	0.6	0.9
(Signal protein)										
VEGF		Piriform	0.9	0.7	0.9	0.5	0.9	1.1	0.9	0.6
		Hippocampus	0.8	0.7	0.6	0.5	0.9	0.9	0.7	0.7
		Neocortex	0.7	0.5	0.7	0.6	0.6	0.7	0.9	0.9
		Cerebellum	0.3	0.7	0.5	0.7	0.5	0.5	0.6	0.7

## Results

The results revealed significant increases in the concentration of numerous cytokines at different time-points in several of the brain regions examined (Tables 1 and 2).

**Table 2:** Cytokine concentration ratio of SE/control animals

Analytes		Brain region	2 h	6 h	24 h	5 days
Cytokines	Chemokines					
	CCL2	Piriform	3**	9**	186**	6**
		Hippocampus	4*	18**	108**	6**
		Neocortex	7**	27**	252**	4
		Cerebellum	1	1	1	1
	CCL3	Piriform	60**	18**	228**	54**
		Hippocampus	69**	25**	211**	238**
		Neocortex	35**	25**	272**	86**
		Cerebellum	1	1	1	1
	CCL5	Piriform	1	1	2**	2*
		Hippocampus	1	1	2*	1
		Neocortex	1	1	2*	1
		Cerebellum	1	1	1	1
IL-1β		Piriform	2*	1	2*	1
		Hippocampus	2*	2*	1	1
		Neocortex	2*	2*	2**	1
		Cerebellum	1	1	1	1
TNF		Piriform	1	1	1/2*	1
		Hippocampus	1	1	1/2*	2*
		Neocortex	1	2	2	1
		Cerebellum	1	1	1	1
Signal protein						
VEGF		Piriform	1	1	1	1
		Hippocampus	1	1	1	1
		Neocortex	1	1	1	1
		Cerebellum	1	1	1	1

*p < 0.05; **p < 0.001

**Fig. 1:**
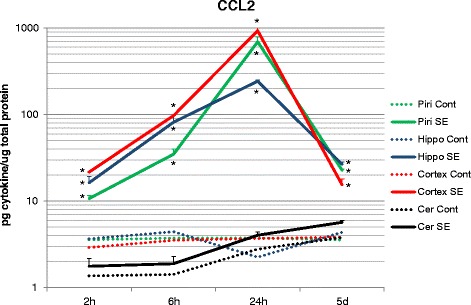
CCL2 concentrations presented with a log scale to illustrate the magnitude of increased CCL2 relative to the other cytokines examined. An increase of 3–7 times the basal levels was observed 2 h after SE, 10–25 times the control levels 6 h after SE, and reached a peak 24 h after SE in the hippocampus, piriform cortex, and neocortex with increases of ~100-, ~200-, and ~250-fold, respectively. Five days after seizures, CCL2 concentration was still significantly elevated in the piriform cortex, hippocampus, and neocortex (*p < 0.001 in all cases). No significant alterations to CCL2 concentrations were observed in the cerebellum

Tissue concentration of CCL2 was significantly increased in seizure animals at all time-points, and in all brain regions, except in the cerebellum. Concentrations peaked at 24 h after SE in the piriform cortex hippocampus, and midline neocortex. Five days after seizures, CCL2 levels were still significantly elevated in the piriform cortex, hippocampus, and neocortex but had declined from the 24-h peak levels (Fig. 1).

**Fig. 2:**
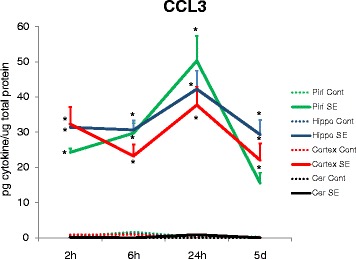
CCL3 concentrations presented an increase of 60 times the basal levels 2 h after SE, 25 times the control levels 6 h after SE, and reached a peak 24 h after SE with a ~250-fold increase in the hippocampus, piriform cortex, and neocortex. Five days after seizures, CCL3 concentration was still significantly elevated in the piriform cortex (~50-fold), hippocampus (~250-fold), and neocortex (~100-fold; *p < 0.001 in all cases). No significant alterations were observed in the cerebellum

The chemokine CCL3 presented a larger increase at earlier time-points relative to CCL2 protein observed 2 h after SE in the piriform cortex, hippocampus, and midline neocortex (Fig. 2). At 6 h after SE, increase in the same areas was followed by a peak concentration 24 h after SE in all regions, except the cerebellum. Five days after SE, concentrations of CCL3 remained significantly elevated in the hippocampus, piriform cortex, and midline neocortex, but to a lesser extent than at the 24-h peak (Fig. 2).

**Fig. 3:**
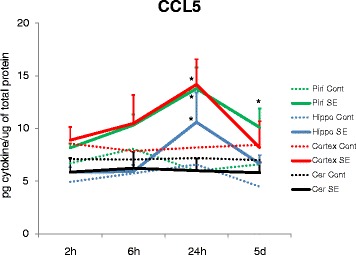
CCL5 concentrations presented a significant twofold increase in the piriform cortex, hippocampus (*p < 0.001), and neocortex (*p < 0.05) 24 h after SE. Five days after seizures, CCL5 concentration was still significantly elevated in the piriform cortex (*p < 0.05). No significant alterations were observed in the cerebellum

Concentration of CCL5 was significantly elevated at 24 h after SE in the piriform cortex and neocortex. No significant alterations were observed in either the hippocampus or the cerebellum. Five days after SE, the CCL5 concentration was still significantly elevated in the piriform cortex but returned close to control levels in other regions (Fig. 3).

**Fig. 4:**
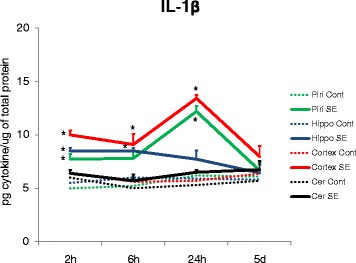
A significant increase in IL-1β concentration was observed in the piriform cortex, hippocampus, and neocortex 2 h after SE (*p < 0.05) and in the hippocampus and neocortex 6 h after SE (*p < 0.05), but not in the cerebellum. The peak concentration was observed 24 h after SE with a ~2-fold increase in the piriform cortex (*p < 0.05) and a ~2.5-fold in the neocortex (*p < 0.001) with no significant alteration in the hippocampus. Five days after seizures, IL-1β levels returned to control levels in all brain regions

The concentration of IL-1β was significantly elevated 2 h after SE in the neocortex, piriform cortex, and hippocampus, but not in the cerebellum (Fig. 4). Concentrations of IL-1β were significantly elevated 6 h after SE in the neocortex and hippocampus, but not in the piriform cortex or cerebellum (Fig. 4). The peak concentration was observed 24 h after SE in the piriform cortex and neocortex. Five days after seizures, IL-1β levels returned to levels similar to control rats in all brain regions (Fig. 4).

Analysis of TNF-α levels revealed significant concentration decrease in the piriform cortex and hippocampus 24 h after SE and a significant concentration increase in the hippocampus 5 days after SE (Table 1). No significant alterations in VEGF levels were observed at any of the time-points, nor in any of the regions analyzed (Tables 1 and 2).

## Discussion

Simultaneous analysis of multiple cytokines in different brain regions, at different time-points after SE, revealed significant alterations in the concentration of CCL2, CCL3, CCL5, IL-1β, and TNF-α. CCL2 and CCL3 showed the largest concentration increases in the piriform cortex, hippocampus, and neocortex that peaked at 24 h after SE. CCL5 and IL-1β were also significantly elevated in these brain regions, although the increases were orders of magnitude lower relative to CCL2 and CCL3. These novel data in the rat pilocarpine SE model are consistent with findings in human epileptic patients, where microarray analyses showed upregulation of genes *ccl3* and *ccl4* in surgically removed hippocampus [10]. Similarly, animal studies showed increased CCL2 and CCL3 mRNA that peak 1 day after electrically induced SE [11], and following kainic acid-induced seizures in rats, the gene for *ccl2* was upregulated in entorhinal, perirhinal, posterior piriform cortices and hippocampus, where its expression level was the highest relative to the other genes examined [12]. Therefore, future translational studies can selectively target one or more of these inflammatory proteins in the pilocarpine SE model.

Further highlighting the clinical relevance of these chemokines, elevated CCL2 protein was observed in the temporal lobe and hippocampus from patients with intractable epilepsy [13], and both CCL2 and CCL3 proteins were elevated in temporal lobe epilepsy patients measured with multiplex assay [4]. We have also previously shown that CCL2 and its receptor CCR2 were elevated in the rat hippocampus 5 days after seizures in rats [8], whereas other studies demonstrated increased CCL3 protein within the first 2 h after pilocarpine injection in mice [14]. Results consistent with these findings were also found in other experimental models of temporal lobe epilepsy, such as tetanic stimulation of the hippocampus [11] and following kainic acid-induced seizures [5]. Thus, there is substantial accumulating evidence that changes in these cytokines may play an important role in epileptogenesis and thus may represent viable therapeutic targets.

From a functional perspective, it is possible that CCL2 is involved in direct impairment of neuronal survival in epileptogenic structures. For example, CCL2 activates caspase signaling pathways [15], which can contribute directly to cell death. CCL2 can also influence glia activation and the ensuing inflammation [16], which results in uncoupling of gap junctions between astrocytes and thus a reduced calcium buffering potential by the astrocytic network observed in temporal lobe epilepsy patients [17]. Further evidence in support for direct functional impairment attributed to CCL2 elevation was reported in a study in which expression by astrocytes in transgenic mice impairs synaptic plasticity in the hippocampus [18]. Indeed, a study that inhibited CCL2 secretion with pyrrolidine dithiocarbamate prior to pilocarpine-induced SE demonstrated beneficial outcomes with less microglial activation and decreased neuronal damage [19]. While such experimentation may not be clinically viable, it does provide evidence for the therapeutic potential of targeting CCL2 to improve epileptogenic outcomes.

Targeting the seizure-induced increase in CCR5 may be another putative therapeutic target to ameliorate the epileptogenic progression. The chemokine CCL5 is chemotactic to T cells, eosinophils, and basophils. Consistent with the findings in the present study, there is an upregulation of CCR5 expression after kainic acid-induced seizures especially in hippocampal formation neurons and glial cells [20] and after pilocarpine-induced seizures in hippocampal astrocytes [21]. Interestingly, the use of RNA interference to lower CCR5 expression in rats protected against kainic acid-induced seizures by decreasing hippocampal neuronal loss and macrophage/microglial infiltration [22]. The fact that CCR5-knockout mice did not show the same protection against inflammatory hippocampal injury suggests that compensatory mechanisms may render it necessary to target multiple inflammatory molecules to achieve optimal benefit [23].

The result of elevated IL-1β following SE is consistent with previous studies showing that increased concentration of IL-1β after seizures exerts a pro-epileptogenic action, and blocking of IL-1β presented anticonvulsive effects while exogenous application of the cytokine worsened electrographic seizure development [2, 4]. In contrast, the results from the current study were inconsistent with previous reports of increased VEGF after pilocarpine-induced seizures [24]. One possible explanation for this discrepancy is that the current study examined total VEGF protein in brain homogenates, whereas the latter study observed elevated cellular VEGF in neurons and astrocytes [24]. Thus, it is possible that the altered VEGF expression observed is a qualitative rather than a quantitative one.

## Conclusions

The data from this short report shows, for the first time, elevated CCL2 and CCL3, as well as increased CCL5, IL-1β, and differentially altered TNF-α. It is becoming increasingly clear that the neuroinflammatory response after an initial precipitating injury such as SE plays an important role in nervous tissue pathology. Understanding the temporal and spatial expression patterns of the neuroinflammatory milieu is essential to develop improved therapeutic interventions following these types of injury. Therefore, simultaneous measurement of multiple cytokines from different brain regions is especially important to elucidate the extensive, intertwined, and complex interactions among the inflammatory proteins. Such studies will enable subsequent studies that target multiple cytokine/chemokines at different time-points and possibly different brain regions to achieve optimal therapeutic efficacy.

## Abbreviations

CCL: C-C motif chemokines
CCL2/MCP-1: monocyte chemoattractant protein-1
CCL3/MIP-1α: macrophage inflammatory protein 1 alpha
CCL5/RANTES: regulated upon activation normal T cell expressed
CCR: CCL receptor
IL: interleukin
TNF: tumor necrosis factor
VEGF: vascular endothelial growth factor
